# Effects of the Bacterial Extract OM-85 on Phagocyte Functions and the Stress Response

**DOI:** 10.1155/S0962935194000189

**Published:** 1994

**Authors:** S. Baladi, S. Kantengwa, Y. R. A. Donati, B. S. Polla

**Affiliations:** Allergy Unit University Hospital 1211 Geneva 14 Switzerland

## Abstract

The effects of the bacterial extract OM-85 on the respiratory burst, intracellular calcium and the stress response have been investigated in human peripheral blood monocytes from normal donors. Activation of the respiratory burst during bacterial phagocytosis has been previously associated with heat shock/stress proteins synthesis. Whereas OM-85 stimulated superoxide production and increased Ca^2+^ mobilization, it fared to induce synthesis of classical HSPs. The lack of stress protein induction was observed even in the presence of iron which potentiates both oxidative injury and stress protein induction during bacterial phagocytosis. However OM-85 induced a 75–78 kDa protein, which is likely to be a glucose regulated protein (GRP78), and enhanced intracellular expression of interleukin-lβ precursor.


Mediators of Inflammation 3, 143–148 (1994)

### Effects of the bacterial extract OM-85 on phagocyte functions and the stress response

S. Baladi, S. Kantengwa, Y. R. A. Donati and B. S. Polla

Allergy Unit, University Hospital, 1211 Geneva 14, Switzerland

CA Corresponding Author

**The effects of the bacterial extract OM-85 on the respiratory burst, intracellular calcium and the stress response have been investigated in human peripheral blood monocytes from normal donors. Activation of the respiratory burst during bacterial phagocytosis has been previously associated with heat shock/stress proteins synthesis. Whereas OM-85 stimulated superoxide production and increased $Ca^{2+}$ mobilization, it failed to induce synthesis of classical HSPs. The lack of stress protein induction was observed even in the presence of iron which potentiates both oxidative injury and stress protein induction during bacterial phagocytosis. However OM-85 induced a 75–78 kDa protein, which is likely to be a glucose regulated protein (GRP78), and enhanced intracellular expression of interleukin-1β precursor.**

**Key words**: Bacterial extract OM-85, Glucose regulated proteins, Heat shock proteins, Interleukin-1β, Intracellular calcium, Oxidative stress, Respiratory burst

### Introduction

Heat shock/stress proteins (HSPs) are a set of conserved proteins induced in virtually all cells and tissues by a number of cellular injuries, including exposure to elevated temperatures, reactive oxygen species (ROS), and phagocytosis of selected cells and/or pathogens.[1] HSPs function as molecular chaperones and participate in translocation, folding and refolding of nascent peptides or proteins altered by the above mentioned conditions.[2] They protect cells from the adverse conditions associated with protein denaturation and exert important survival functions in stressed cells.[3]

Stress proteins are usually named according to their apparent molecular weight and classified into families according to their respective inducers. The glucose regulated proteins (GRPs) are members of those families induced by glucose deprivation or alterations in calcium metabolism leading to an increase or decrease in cytosolic free calcium as induced, for example, by calcium ionophores.[4] Heme oxygenase is also part of the stress protein families; in human cells it is an oxidation specific stress protein and may function as a radical scavenger.[5,6]

Although universal, the stress response is modulated by cell differentiation and appears particularly complex in human monocytes–macrophages.[7,8] In these cells, we have shown that phagocytosis of *Staphylococcus aureus* induces the synthesis of HSP70.[9] This induction appeared tightly linked to the activation of the respiratory burst enzyme NADPH oxidase and the generation of superoxide anion ($O_2^-$) during phagocytosis. We suggest that the induction of HSP70 during bacterial phagocytosis may trigger protective functions within phagocytes against the toxic products produced during phagocytosis, in particular, ROS.[10]

Bacterial extracts have been used extensively for the prevention of recurrent infections of the upper respiratory tract.[11] The immunomodulator Broncho-Vaxom (OM-85) modulates cellular functions such as cytokine production and activation of natural killer cells.[12–14] OM-85 increases respiratory burst and bacterial killing by polymorphonuclear (PMN) cells.[15] OM-85 also increased $Ca^{2+}$ mobilization.[15]

We investigated the effects of exposure of human phagocytes to OM-85 on respiratory burst, cytosolic calcium levels and protein synthesis, addressing in particular whether OM-85 induces HSP synthesis, and whether such induction is linked to the activation of NADPH oxidase. We found that OM-85 directly activated NADPH oxidase in human monocytes but, in contrast to phagocytosis of whole bacteria, did not induce the synthesis of the classical stress proteins in these cells.

### Materials and Methods

*Cell preparation and culture:* Peripheral human mononuclear cells from healthy volunteers were isolated by Ficoll gradient and monocytes purified by adherence. Mononuclear cells were incubated for 45 min in antibiotic-free RPMI 1640 medium (Gibco Laboratories, Paisley, Scotland) supplemented with 10% foetal calf serum (FCS, Gibco) and 1% glutamine

© 1994 Rapid Communications of Oxford Ltd

(Gibco) (complete medium, CM), then washed four to six times with phosphate buffered saline (PBS, Gibco), to remove the lymphocytes. Monocytes were then further incubated in CM for superoxide production experiments or in CM without methionine for protein synthesis analysis. Incubation was overnight, at 37°C in a humidified atmosphere containing 95% air and 5% $CO_2$. Neutrophils were prepared as described previously, from blood obtained from normal volunteers.[16]

*Preparation of OM-85 and analysis of LPS content:* OM-85 is a standardized bacterial extract obtained by controlled alkaline hydrolysis of different strains of bacteria—*Staphylococcus aureus, Streptococcus pyogenes* and *viridans, Diplococcus pneumoniae, Klebsiella ozaenae, Klebsiella pneumoniae, Neisseria catarrhalis* and *Haemophilus influenzae*. Due to the prolonged action of alkali, bacterial proteins are modified and this generates acidic polypeptides (7 mg/protein/ml according to Lowry, 23 mg dry weight/ml). The low endotoxin content (0.3 μg/ml) as measured by the *Limulus* ameobocyte lysate assay (LAL, Haemachem Inc., St Louis, MO)[17] also relates to the prolonged action of alkali which is known to detoxify LPS by hydrolysis of specific fatty acids.[18] OM-89, another bacterial extract used as a comparison in our experiments, was obtained by controlled alkaline hydrolysis of 18 different *Escherichia coli* strains (endotoxin content higher than OM-85 but less than 2 μg/ml).

*Protein analysis:* Cells were incubated with different concentrations of OM-85 (or OM-89) for 3 h or 16 h. Heat shock (positive control) was performed by incubating the relevant culture plates in a water bath at 44°C for 20 min. The heat-shocked cells were allowed to recover at 37°C for 2 h before labelling. All cells were labelled with 6 μCi/ml ([$^{35}$S]-methionine (specific activity >1 000 Ci/mmol) (Amersham, Buckinghamshire, UK) for 90 min. Cells were then harvested, washed twice with PBS, lysed in sodium dodecyl sulphate (SDS) sample buffer with 4% mercapto-ethanol and boiled for 10 min. Samples adjusted for [$^{35}$S]-methionine incorporation were electrophoresed on SDS-polyacrylamide gels according to Laemmli[19] (10% polyacrylamide). The proteins were revealed by autoradiography on X-omat AR films (Kodak, Lausanne, Switzerland).

*Immunoblotting analysis:* Proteins from aliquots corresponding to equal cell numbers were electrotransferred to nitrocellulose membranes. The membranes were saturated with casein-containing buffer for 2 h and then hybridized with the appropriate antibodies, i.e. a monoclonal anti-inducible HSP70 (Stress Gen), a polyclonal anti-IL-1β (Genzyme), and various anti GRP78 (mAb 7.10 and 5A5, kind gifts from Susan Lindquist (University of Chicago) and Susan Pierce (Northwestern University, Evanston) respectively, or the commercial antibody MA3-001 from Affinity BioReagents, (Neshanic Station, NJ). Proteins of interest were detected as described.[9]

*Superoxide production:* Superoxide production was measured by the superoxide dismutase inhibitable reduction of ferricytochrome C as described previously.[20] Zymosan was opsonized with human serum as described.[20] Cells were washed twice with PBS then incubated in serum free medium for 30 min and stimulated with either PMA, 100 ng/ml, or opsonized zymosan (3 mg/ml) (positive controls) or with increasing amounts of bacterial extracts.

*Measurements of cytosolic free calcium:* Cells (20 × $10^6$ cells) were loaded with fura-2 AM at 37°C for 30 min. Prior to measurement fluorescence, cells were washed and resuspended at 8 × $10^6$ cells/ml in buffer containing 136 mM NaCl, 5 mM KCl, 1.2 mM $MgSO_4$, 1.2 mM $KH_2PO_4$, 5 mM $NaHCO_3$, 20 mM Hepes, 5.5 mM glucose, 0.2 mM EDTA, 1.2 mM $CaCl_2$; pH 7.4. Fluorescence was measured in a Perkin–Elmer spectrofluorimeter at an excitation wavelength of 340 nm and at 510 nm emission wavelength. $[Ca^{2+}]_i$ was calculated using the following expression: $[Ca^{2+}]_i = K_d[(F - F_{min})/(F_{max} - F)]$. $K_d$ was assumed to be 224 nM at 37°C.[21]

### Results

*Effects of the OM-85 on the respiratory burst:* OM-85 stimulated the respiratory burst enzyme NADPH oxidase in human peripheral blood monocytes in a dose dependent manner. One representative experiment (out of six) is shown in Fig. 1. This effect was also observed with OM-89, but to a lesser degree (data not shown) which is of relevance when consid-

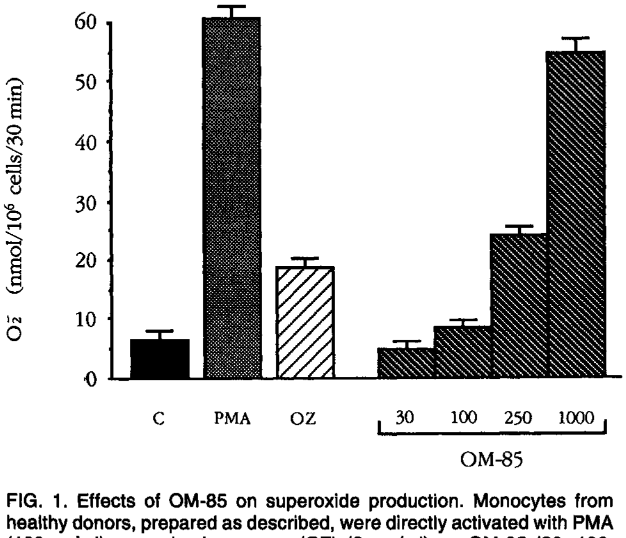

FIG. 1. Effects of OM-85 on superoxide production. Monocytes from healthy donors, prepared as described, were directly activated with PMA (100 ng/ml), opsonized zymosan (OZ) (3 mg/ml), or OM-85 (30, 100, 250, or 1 000 μg/ml) for 30 min. Superoxide production was measured as described and expressed in nmol/$10^6$ cells. Data are from one representative experiment out of six. Bars represent means ± S.E.M., $n$ = 3.

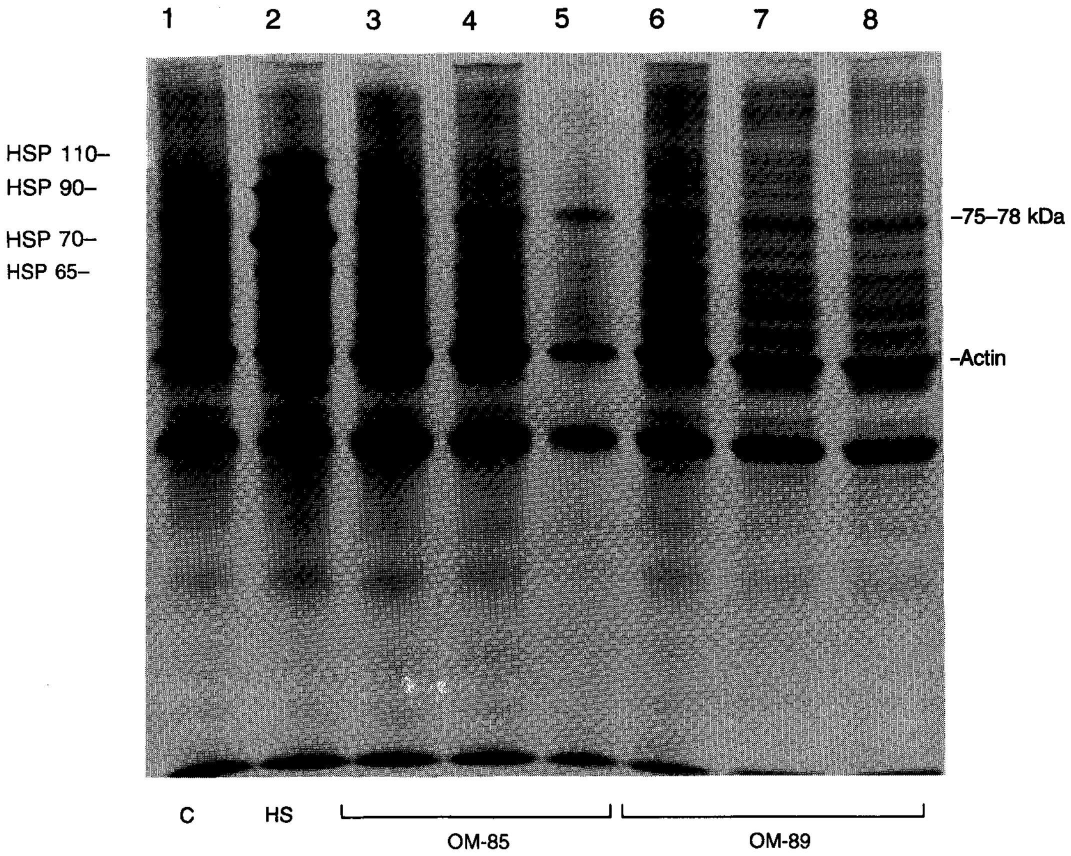

FIG. 2. Effects of OM-85 and OM-89 on protein synthesis in human monocytes. Monocytes were incubated with increasing amounts of OM-85 (30, 100, 250 µg/ml) (Lanes 3–5) or OM-89 (30, 100, 250 µg/ml) (Lanes 6–8) for 16 h. Proteins were labelled with $^{35}$S-methionine for 90 min and analysed by SDS–PAGE. The autoradiogram shows the protein of 75–78 kDa in the samples treated with the bacterial extracts. Lane 1, cells maintained at 37°C; Lane 2, cells exposed to 44°C for 20 min. Similar results were obtained for shorter incubations (3 h).

ering the respective LPS content of the two extracts. Furthermore, LPS by itself (tested at 0.1 and 0.2 µg/ml) did not activate NADPH oxidase in human monocytes (our unpublished data) further arguing against a nonspecific effect of LPS contained in the bacterial extracts on the respiratory burst.

In contrast, when cells were preincubated overnight with OM-85 and then activated with either PMA or opsonized zymosan, there was no priming effect on human peripheral blood monocytes for superoxide production (not shown). This latter result was also observed when using OM-89.

*Effects of OM-85 on protein synthesis:* The bacterial extracts did not induce the synthesis of HSP70 or any of the classical HSPs. Fig. 2 shows one of more than 20 experiments using biometabolic labelling. The lack of HSP70 induction was further established by using the more sensitive technique of Western blotting with monoclonal antibody against the inducible HSP70 (Fig. 3). When OM-85 was used at very high concentrations (1–2 mg/ml), there was, in some experiments, an inhibition of normal protein synthesis (Fig. 2, Lane 5), likely related to the high production of ROS and their well-known toxic effects on protein synthesis.[22–24] Even under these conditions however (i.e. under conditions associated with a decrease in normal protein synthesis), there was still no induction of HSP70. We have previously reported that during phagocytosis of *S. aureus*, iron poten-tiated the induction of HSP70 and induced the synthesis of heme oxygenase, probably by catalysing the production of highly reactive hydroxyl radicals via the Fenton reaction.[9] In the experiments described here however, neither HSP70 nor heme oxygenase were induced even in the presence of iron (Fig. 4), indicating that the generation of ROS induced by

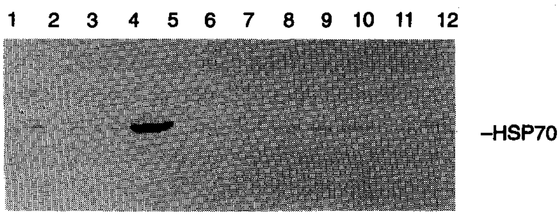

FIG. 3. Western blot analysis of HSP70 in monocytes exposed to OM-85, OM-89 and/or A23187 using a monoclonal antibody against the human inducible HSP70. Monocytes were exposed to A23187 (7 µM) (Lane 3); 44°C, 20 min (Lane 4); 100 µg/ml OM-89 in the absence (Lane 5) or the presence (Lane 6) of A23187; 250 µg/ml OM-89 without (Lane 7) or with (Lane 8) A23187; 100 µg/ml OM-85 alone (Lane 9) or in combination with A23187 (Lane 10); 250 µg/ml OM-85 without (Lane 11) or with (Lane 12) A23187. Incubation was for 3 h at 37°C. Unstressed cells are shown in Lane 1 and cells incubated with DMSO, the vehicle for A23187, in Lane 2.

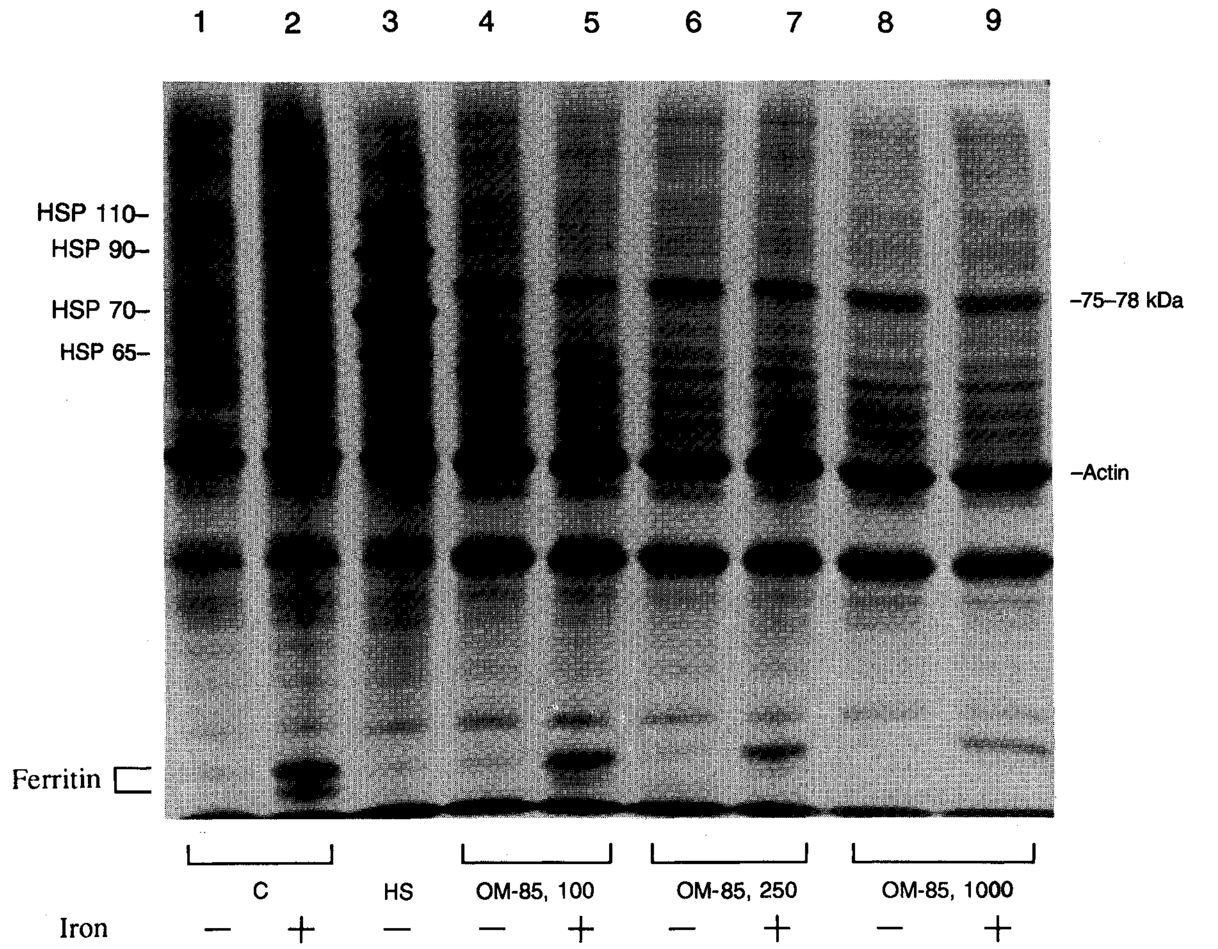

FIG. 4. Effects of OM-85 on protein synthesis in the presence of iron. Monocytes were exposed to $Fe^{2+}$ (500 μM) (Lane 2); 44°C, 20 min (Lane 3); 100 μg/ml OM-85 in the absence (Lane 4) or the presence of $Fe^{2+}$ (Lane 5); 250 μg/ml OM-85 without (Lane 6) or with $Fe^{2+}$ (Lane 7); 1 mg/ml without (Lane 8) or with $Fe^{2+}$ (Lane 9). Lane 1, control cells.

OM-85 was insufficient, even in the presence of iron, to induce oxidation specific stress proteins.

In contrast, incubation of monocytes with OM-85 (30, 100, 250 μg/ml) as well as with OM-89 (30, 100, 250 μg/ml) induced in these cells the synthesis of a number of proteins (Fig. 2), and in particular of a 75–78 kDa protein which, according to molecular weight and induction pattern might be GRP78. However, attempts to characterize this protein with various antibodies raised against GRP78 were inconclusive.

Furthermore, we observed a 36 kDa protein, which was induced or increased to various degrees depending upon its constitutive expression in the different experiments. This protein was identified as the intracellular precursor of interleukin-1ß by immunoblotting with anti-IL-1ß antibody (Fig. 5).

*Effects of OM-85 on cytosolic free calcium:* GRP78 is a member of the glucose-regulated stress protein families specifically involved in the handling of abnormal proteins within the endoplasmic reticulum, and induced by alterations in intracellular calcium. In order, therefore, to unravel the mechanism of selective induction of the putative GRP78 by OM-85, we

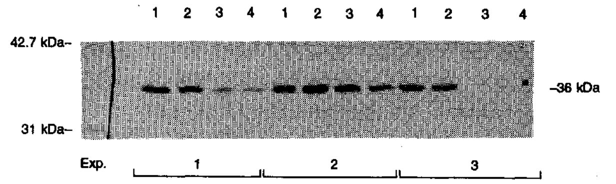

FIG. 5. OM-85 induces intracellular interleukin-1. Monocytes from healthy donors were incubated with OM-89 (5 μg/ml) (Lane 2, Exp. 1); OM-89 (100 μg/ml) (Lane 2, Exp. 2); OM-89 (1 mg/ml) (Lane 2, Exp. 3); OM-85 (5 μg/ml) (Lane 1, Exp. 1); OM-85 (100 μg/ml) (Lane 1, Exp. 1); OM-85 (1 mg/ml) (Lane 1, Exp. 3). For each experiment, Lane 4 is control; Lane 3 is HS).

investigated whether OM-85 induced a rise in $[Ca^{2+}]_i$ in monocytes. Fura-2 AM-loaded monocytes were stimulated by 375 μg/ml of OM-85 or OM-89 and cytosolic $Ca^{2+}$ was measured. Both bacterial extracts were able to increase $[Ca^{2+}]_i$, but, as for superoxide production, OM-85 was more effective (Fig. 6). These results are in good agreement with previous reports on $Ca^{2+}$ mobilization by OM-85 and OM-89 in PMN cells[15] and support the hypothesis that the 78 kDa doublet induced by these agents could be GRP78.

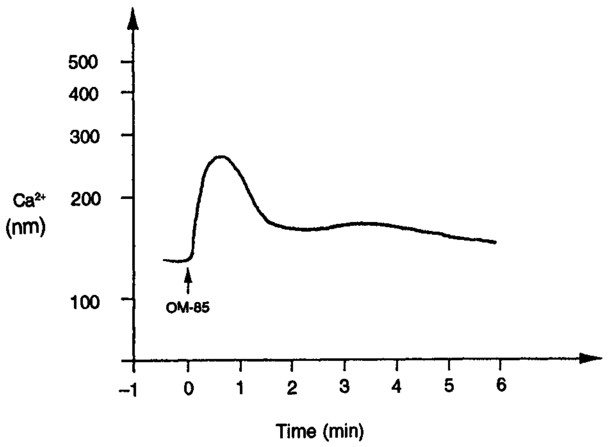

FIG. 6. OM-85 induces a rapid and reversible rise in cytosolic free calcium in human monocytes. Monocytes were loaded with Fura-2 AM as described. The rise in $[Ca^{2+}]_i$ after exposure to increasing concentrations (0.4 mg/ml to 1.6 mg/ml) of OM-85 was measured on $8 \times 10^6$ cells. As the $[Ca^{2+}]_i$ peak showed minimal dose-dependence with the OM-85 concentration tested, only the response to the lowest dose (0.4 mg/ml) is presented here.

### Discussion

The results of this study establish that the bacterial extract OM-85 is able to modulate metabolic functions in monocytes. Indeed we observed that OM-85 and, to a lesser extent, OM-89 activate the respiratory burst in human monocytes (Fig. 1), as well as in neutrophils (our unpublished data and Reference 15). This activation appeared to result from a direct effect on the enzyme NADPH oxidase since pre-incubation with both extracts failed to prime the cells for further stimulation. OM-85 thus appears unique as a compound able to directly activate NADPH oxidase, but without resulting in the priming of the enzyme, which is in contrast with a number of other compounds, including phorbol esters, calcium ionophores or tumour necrosis factor-α. Activation of phagocytes by OM-85 for $O_2^-$ generation may contribute to the host's anti-bacterial defences as reported previously.[25] In agreement with our control experiments Bottex *et al.*[26] also showed that the stimulatory activity of OM-85 is not due to residual contamination with LPS. In contrast, the fact that OM-85, as well as OM-89, failed to prime monocytes for $O_2^-$ production suggests that these products have no pro-inflammatory activity, at least with respect to the involvement of ROS in inflammation.

Since heat shock proteins are markers of a variety of cellular stresses, we investigated the effects of the bacterial extracts on protein synthesis in human monocytes. OM-85 induced neither classical stress proteins nor the oxidative stress specific heme oxygenase even under conditions where the synthesis of total protein was reduced (see Fig. 2, Lane 5). Characterization of the most abundant heat shock protein HSP70 by the more sensitive immunoblotting technique confirmed the absence of this inducible protein (Fig. 3). Similar results were obtained with OM-89. We have previously reported that phagocytosis of whole *S. aureus* induced in monocytes the synthesis of HSP70. This induction was enhanced and heme oxygenase was induced in the presence of iron.[9] With the bacterial extracts, however, and even with the potent activator of $O_2^-$ production, OM-85, iron failed to increase HSP70 and heme oxygenase synthesis (Fig. 4).

The lack of induction of HSP70 by OM-85 indicates that this agent does not lead to the type of cellular toxicity which in turn will induce a stress response. Furthermore, since the induction of heme oxygenase is dependent upon the intracellular generation of the highly reactive hydroxyl radicals, the lack of such induction by OM-85 indicates that the generation of ROS, whether extracellular or intracellular, induced by the bacterial extract is insufficient to induce oxidation specific stress proteins, even in the presence of iron which catalyses ·OH production. The induction of $O_2^-$ production by OM-85 may be essentially extracellular, which would explain the lack of oxidative modifications of intracellular proteins—the classical signal for stress protein induction—and therefore the lack of increased synthesis of the classical HSPs.

OM-85 induced a protein of 75–78 kDa (Figs 2 and 4) which appears to be related to the glucose-regulated protein GRP78 (BiP). Similar results again were obtained with OM-89. However, attempts to characterize this protein with available antibodies against GRP78 were unsuccessful. We are currently performing experiments aimed at the definitive identification of GRP78 using appropriate molecular probes.

Among the inducers of GRP78 are agents stimulating calcium mobilization such as calcium ionophore A23187. Therefore we measured intracellular calcium in monocytes exposed to OM-85. As shown in Fig. 6, there was a rapid increase in $[Ca^{2+}]_i$ after addition of the bacterial extracts. These results are in good agreement with those reported in neutrophils by Nauck *et al.*[15] The rise in $[Ca^{2+}]_i$ induced by the bacterial product along with the 75–78 kDa protein further supports the possibility that this protein is indeed GRP78. OM-89 also induced a rise in $[Ca^{2+}]_i$, although much smaller than OM-85 (unpublished data). The parallelism between these two effects of the bacterial extracts (on NADPH oxidase activation and on $[Ca^{2+}]_i$) suggests a receptor mediated event rather than an effect relating to protein kinase C mediated phosphorylation.[27]

Another protein of 35–36 kDa was either increased or induced by OM-85, depending on its basal expression in the monocytes of a given blood donor. By immunoblotting, this protein has been recognized by a polyclonal antibody against IL-1β, suggesting that it is the cytokine precursor. Previous reports have demonstrated the effects of Broncho–Vaxom on cytokine production in normal subjects.[28] Our data indicate that increased production is associated with increased intracellular synthesis of the cytokine and further support the view that the bacterial extracts studied are indeed immunomodulators, at least under the *in vitro* conditions examined here. Given the concentrations of endotoxins within the bacterial extracts, the synthesis of IL – 1β is unlikely to be due to endotoxins. Indeed, as was the case for NADPH oxidase activation, it has been established previously that whereas both OM-85 and LPS activate IL-1 secretion, this effect was abolished when heating the bacterial extract, which was not the case with LPS.[26]

In conclusion, our data suggest that OM-85, which directly activates the respiratory burst enzyme NADPH oxidase, but without priming, represents a safe therapeutic product in promoting antibacterial activity, without additional pro-inflammatory effect. As suggested by the lack of induction of a classical stress response, NADPH activation by OM-85 does not lead to oxidation induced protein modifications, which would represent the ultimate common signal for stress protein induction. Our results are in good agreement with previous reports[29–31] showing that OM-85 behaves as an immunomodulator in human monocytes, but not as a stress protein inducer.

### References

1. Polla BS, Perin M, Pizurki L. Regulation and functions of stress proteins in allergy and inflammation. *Clin Exp Allergy* 1993; **23**: 548–556.
2. Young DB. Chaperonins and the immune response. *Sem Cell Biol* 1990; **1**: 27–35.
3. Welch WJ. The mammalian heat shock (or stress) response: a cellular defence mechanism. *Adv Exp Med Biol* 1987; **225**: 287–304.
4. Welch WF, Garrels JG, Thomas GP, Lin JJ, Feramisco JR. Biochemical characterization of the mammalian stress proteins as glucose and $Ca^{2+}$ ionophore regulated proteins. *J Biol Chem* 1983; **258**: 7102–7111.
5. Keyse SM, Tyrrell RM. Heme oxygenase is the major 32-kDa stress protein induced in human skin fibroblasts by UVA radiation, hydrogen peroxide, and sodium arsenite. *Proc Natl Acad Sci USA* 1989; **86**: 99–103.
6. Polla BS, Mariéthoz E, Kantengwa S. Differential regulation of heat shock proteins and heme oxygenase during phagocytosis. *American Oil Chemists' Society* (in press).
7. Donati YRA, Kantengwa S, Polla BS. Phagocytosis and heat shock response in human monocytes-macrophages. *Pathobiol* 1991; **59**: 156–161.
8. Kantengwa S, Donati YRA, Clerget M, *et al.* Heat shock proteins: an autoprotective mechanism for inflammatory cells? *Sem Immunol* 1991; **3**: 49–56.
9. Kantengwa S, Polla BS. Phagocytosis of *Staphylococcus aureus* induces a selective stress response in human monocytes-macrophages (Mø): modulation by Mø differentiation and by iron. *Infect Immun* 1993; **61**: 1281–1287.
10. Polla BS, Mili N, Kantengwa S. Heat shock and oxidative injury in human cells. In: Maresca B, Lindquist S, eds. *Heat Shock*. Berlin, Heidelberg: Springer-Verlag, 1991; 279–290.
11. Paupe J. Immunotherapy with an oral bacterial extract (OM-85 BV) for upper respiratory infections. *Respiration* 1991; **58**: 150–154.
12. Cvoriscec B, Ustar M, Pardon R, Palecek I, Stipic-Markovic A, Zimic B. Oral immunotherapy of chronic bronchitis: a double-blind placebo-controlled multicentre study. *Respiration* 1989; **55**: 129–135.
13. Keller R, Hinz G. Effect of an oral polyvalent bacterial lysate (Broncho-Vaxom) in chronic bronchitis. *Prax Klin Pneumol* 1984; **38**: 225–228.
14. Wybran J, Libin M, Schandene L. Activation of natural killer cells and cytokine production in man by bacterial extracts. *Immunopharmacol Immunotoxicol* 1989; **11**: 17–32.
15. Nauck M, Matthys H, Emmons LR, *et al.* The immunomodulators Broncho-Vaxom and Uro-Vaxom stimulate the bacterial killing and oxidative metabolism of polymorphonuclear leukocytes by the activation of phosphatidylinositol turnover. *Int J Exp Clin Chemotherapy* 1991; **4**: 1–11.
16. Maridonneau-Parini I, Tringale JN, Tauber AI. Identification of distinct activation pathways of the human neutrophil NADPH oxidase. *J Immunol* 1986; **137**: 2925–2930.
17. Levin J, Bang FB. The role of endotoxin in the extracellular coagulation of *Limulus* blood. *Bull Johns Hopkins Hosp* 1964; **115**: 265–274.
18. Somlyo B, Csanky E, Shi XM, *et al.* Molecular requirements of endotoxin (ET) actions: changes in the immune adjuvant, TNF liberating and toxic properties of endotoxin during alkaline hydrolysis. *Int J Immunopharmac* 1992; **14**: 131–142.
19. Laemmli UK. Cleavage of structural proteins during the assembly of the head of bacteriophage T4. *Nature (London)* 1970; **227**: 680–685.
20. Polla BS, Werlen G, Clerget M, Pittet D, Rossier MF, Capponi A. 1,25-dihydroxyvitamin $D_3$ induces responsiveness to the chemotactic peptide f-met-leu-phe in the human monocytic line U937: dissociation between calcium and oxidative metabolic responses. *J Leuk Biol* 1989; **45**: 381.
21. Grynkiewicz G, Poenie M, Tsien RY. A new generation of $Ca^{2+}$ indicators with greatly improved fluorescence properties. *J Biol Chem* 1985; **260**: 3440–3450.
22. Jornot L, Mirault ME, Junod AF. Protein synthesis in hyperoxic endothelial cells: evidence for translational defect. *J Appl Physiol* 1987; **63**: 457–464.
23. Junod AF, Clément A, Jornot L, Peterson H. Differential effects of hyperoxia and hydrogen peroxide on thymidine kinase and adenosine kinase activities of cultured endothelial cells. *Biochim Biophys Acta* 1985; **847**: 20–24.
24. Polla BS, Healy AM, Wojno WC, Krane SM. Hormone 1α,25-dihydroxyvitamin $D_3$ modulates heat-shock response in monocytes. *Am J Physiol* 1987; **252**: C640–C649.
25. Mauel J, Pham TV, Kreis B, Bauer J. Stimulation by a bacterial extract (Broncho-Vaxom) of the metabolic and functional activities of murine macrophages. *Int J Immunopharmac* 1989; **11**: 637–645.
26. Bottex C, Cristau B, Corazza JL, Mougin B, Fontanges R. Effects of two bacterial extracts, OM-85 and Broncho-Vaxom, on IL-1 release and metabolic activity of a murine macrophage cell-line. *Int J Immunotherapy* 1988; **IV**: 203–212.
27. Maridonneau Parini I, Clerc J, Polla BS. Heat shock inhibits NADPH oxidase in human neutrophils. *Biochem Biophys Res Commun* 1988; **154**: 179–186.
28. Wybran J, Libin M, Schandene L. Enhancement of cytokine production and natural killer activity by an *Escherichia coli* extract. *Onkologie* 1989; **12**: 22–25.
29. Bessler WG, Kleine B, Martinez-Alonso C, *et al.* Biological activity of bacterial surface components: bacterial extracts and defined bacterial cell wall components as immunomodulators. *Lung* 1990; **168**(Suppl.): 707–715.
30. Bessler WG, Sedelmeier E. Biological activity of bacterial cell wall components. Immunogenicity of an immunostimulating bacterial extract. *Arzneim-Forsch/Drug Res* 1993; **43**: 502–507.
31. Lusuardi M, Capelli A, Carli S, Spada EL, Spinazzi A, Donner CF. Local airways immune modifications induced by oral bacterial extracts in chronic bronchitis. *Chest* 1993; **103**: 1783–1791.

ACKNOWLEDGEMENTS. The authors are grateful to O.M. for providing the necessary support for these studies. S.B. was supported by the Fonds National de la Recherche Scientifique (FNRS), grant No. 32-028645.90 (to B.S.P.) and S.K. by WHO grant TDR 900538 and FNRS grant No. 32-37464.93 (to B.S.P.).

**Received 15 November 1993; accepted in revised form 4 January 1994**
